# Increased Temperature and Exposure to Ammonium Alter the Life Cycle of an Anuran Species

**DOI:** 10.1002/ece3.70685

**Published:** 2024-12-02

**Authors:** Francisco Javier Zamora‐Camacho, Pedro Aragón

**Affiliations:** ^1^ Department of Biogeography and Global Change Museo Nacional de Ciencias Naturales (MNCN‐CSIC) Madrid Spain; ^2^ Department of Biology of Organisms and Systems University of Oviedo Oviedo Spain

**Keywords:** agrosystem, carryover effects, fertilizer, global change, global warming, natural habitat, *Pelophylax perezi*

## Abstract

Amphibian populations are undergoing a major recession worldwide, likely triggered by global change components such as the global warming and pollutants, among which agrochemicals, in general, and fertilizers, in particular, play a central role given their relevance in agriculture. Potential synergies among these stressors could maximize their individual effects. In this work, we investigated the consequences of a controlled chronic exposure to increased temperature and a sublethal dose of ammonium during the larval stage of *Pelophylax perezi* frogs on the growth, development, and locomotor performance of tadpoles and the metamorphs they gave rise to. To that end, tadpoles were reared either in heated or nonheated tanks, with or without ammonium added. The parents of these tadpoles came from either a pine grove or an agrosystem. Survival was reduced in agrosystem tadpoles reared with ammonium. Increased temperature potentiated tadpole growth while giving way to smaller metamorphs. Faster growth could be a consequence of increased metabolism, whereas the smaller size could follow an accelerated development and metamorphosis, which reduced the growth period. Also, swimming speed was greater in tadpoles reared in heated tanks, while jumping distance was greater in metamorphs reared in nonheated tanks. The effects of temperature were more marked in agrosystem than in pine grove individuals, which could mirror reduced adaptability. Thus, the ability to withstand the effects of these stressors was lower in agrosystem tadpoles.

## Graphical abstract

When chronic exposure to ammonium and artificial heating were applied to tadpoles, tadpoles in heated water were larger, metamorphosed earlier, and as smaller metamorphs. Heated water produced faster larvae but metamorphs reared in nonheated water jumped farther. Ammonium caused increased mortality.

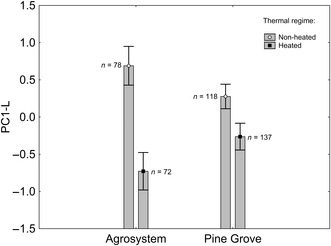

## 1 Introduction

The planet is undergoing a global change triggered by multifarious sources of anthropic pressure (Intergovernmental Panel on Climate Change 2014; Sage 2020). Simply feeding the increasing human population conflicts with the preservation of nature (Cazalis, Loreau, and Henderson 2018) by incurring major changes in land use (Hasan et al. 2020). Along with the plowing of new croplands at the expense of natural areas (Barbier 2020), the shift from extensive to intensive agricultural techniques in the last decades has potentiated inputs of agrochemicals in general (Ganguly et al. 2021) and fertilizers in particular (Conant, Berdanier, and Grace 2013). Fertilizers are known to disrupt nutrient cycles in the ecosystem scale (Gundale 2021) and function as toxicants at the physiological level (Nadarajan and Sukumaran 2021). However, given the complexity of the physical, chemical, and biological interactions between fertilizers deposited and the natural environments, different taxa can be affected through diverse pathways, although most of them lead to bottlenecks with negative effects at various levels, from genetic to populational (Nijssen, WallisDeVries, and Siepel 2017; Sullivan and Sullivan 2017).

Besides these alterations of the land uses and ground composition, human activities also modify the physicochemical characteristics of the atmosphere by releasing greenhouse effect gases responsible for a global warming (Yoro and Daramola 2020). The concomitant increase in temperature has exerted major effects on traits such as phenology (Cohen, Lajeunesse, and Rohr 2018) and chorology (Chen et al. 2011) of multiple taxa, giving rise to unforeseen ecological relationships whose outcome is virtually unpredictable (Traill et al. 2010). Also, the global warming has been accompanied by morphological (Ryding et al. 2021) and physiological changes (Chown et al. 2010) in certain species. Being dependent on external heat sources to regulate their body temperature, ectotherms are the most susceptible animals to the global warming (Jørgensen et al. 2022).

Contamination and the global warming have been suggested to underlie the current amphibian global decline (Hussain and Pandit 2012). Amphibians' cyclic alternation between terrestrial and aquatic environments increases their range of exposure to stressors (Todd et al. 2011; Glinski et al. 2021), while their permeable skins facilitate the access of certain pollutants (Slaby et al. 2019). Amphibians in the terrestrial stage can actively avoid sites that are contaminated (Sievers et al. 2019) or thermally unfavorable (Hoffmann, Cavanough, and Mitchell 2021). Contrastingly, most tadpoles live confined within waterbodies, which are oftentimes relatively small and shallow, thus chemically and thermally homogeneous, forcing the larvae to endure the conditions in them (Céréghino et al. 2008). Therefore, the repercussions of such stressors on larval amphibians merit particular attention.

In the first place, detrimental effects of diverse pollutants (Egea‐Serrano et al. 2012) including fertilizers (Baker, Bancroft, and Garcia 2013) and, more specifically, nitrogenous compounds (Griffis‐Kyle and Ritchie 2007), have been detected on growth and survival of larval amphibians. Among nitrogenous compounds, ammonium is frequently the dominant form in agrosystems (Olivares, Bedmar, and Sanjuán 2013), from where it percolates to nearby waterbodies (Liu et al. 2021). Multiple studies on the effects of ammonium on anuran larvae have revealed major impairments of growth, behavior, or even survival (e.g., Egea‐Serrano, Tejedo, and Torralva 2011; Ilha and Schiesari 2014; Garriga, Montori, and Llorente 2017; Zambrano‐Fernández, Zamora‐Camacho, and Aragón 2022), although these effects may vary among species (Dimitrova and Lukanov 2024). However, many anuran larvae are herbivorous, so that they may benefit from the increase in primary production favored by fertilizers, which may obscure the net effect of fertilizers as pollutants (Egea‐Serrano and Tejedo 2014).

In the second place, being ectotherms, amphibians depend on the range of temperatures available in the environment to remain within their thermal optima (Navas et al. 2021), which can be more or less narrow (Goldstein, von Seckendorr Hoff, and Hillyard 2017). By definition, those thermal optima enhance traits such as growth, development (Peng et al. 2020), locomotion (Herrel and Bonneaud 2012), gut microbiota—on which the efficiency of digestion relies to a great extent (Niu et al. 2023)—or even survival (Dastansara et al. 2017). Likely following genetic differentiation according to thermal clines (Cayuela et al. 2022), thermal biology of tadpoles is frequently adjusted to their climate of provenance (Bonino, Cruz, and Perotti 2020). Higher temperatures contribute to shortening larval stages, which has been corroborated in the wild (Reading 2003) as well as in controlled conditions (Maciel and Juncá 2009). Also, an increase in temperature during the larval stage is also known to affect body proportions of tadpoles (Merilä et al. 2004), and, depending on the magnitude of it, it can lead to reduced growth, development, and even survival (Harkey and Semlitsch 1988; Bellakhal et al. 2014; Turriago, Parra, and Bernal 2015).

Those effects of temperature could be driven by altered gene expression (Fan et al. 2021), hormone secretion (Ren et al. 2021), and metabolism (Kleymenov, Lyapkov, and Ozernyuk 2023), with the concomitant imbalance in the processes governed by them. In this context, the metabolic routes capable of detoxifying agrochemicals such as herbicides (Grott et al. 2022) and pesticides (Quiroga et al. 2024) appear to be impaired at high temperatures. Surprisingly, the potential interactive effects of warming and contamination by fertilizers on amphibian larvae have received scarce attention. Egea‐Serrano and Van Buskirk (2016) induced a single pulse of ammonium nitrate in a short‐term (16‐day) experiment involving *Rana temporaria* tadpoles exposed to two thermal regimes in outdoor mesocosms, where any collateral effects of the fertilizer (such as proliferation of algae on which the tadpoles might feed) or the occurrence of natural denitrification were uncontrolled; no interaction between temperature and the fertilizer was observed.

In this work, we conducted an experiment to examine the interactive effects of temperature and chronic ammonium contamination during the entire larval stage of Iberian green frogs ( *Pelophylax perezi* ) under controlled conditions preventing algal proliferation and denitrification. To that end, eggs were raised until metamorphosis, exposed or not to a sublethal dose of ammonium, in each case either at room temperature or in heated water. The eggs were obtained from parents either from natural habitats or from agroecosystems. We then studied the effects of these treatments on body size, development, and locomotor performance, a common surrogate of whole‐organism condition (Lailvaux and Husak 2017), of larvae and metamorphs. In light of the above, we expected negative effects of contamination and water heating, which we predicted to be exacerbated when both factors were combined (Nowakowski et al. 2018). However, these effects could be milder in agrosystem individuals, if they have evolved some degree of tolerance to the greater pollution (Hua et al. 2017) and temperature (Ellison et al. 2017). In fact, both pollution (Loria, Cristescu, and Gonzalez 2019) and temperature (Muir et al. 2014) may act as selective pressures to which amphibians can become tuned, leading to diverging degrees of tolerance to these stressors.

## 2 Materials and Methods

### 2.1 Study Species

*Pelophylax perezi* is a medium‐sized Ranid widespread in the south of France and most of the Iberian Peninsula. Throughout its distribution range, this euryoecious, semiaquatic frog occupies from clean and fresh to brackish and even contaminated waters and abounds in pristine and human‐altered environments. Reproduction usually takes place in the spring or early summer, when females lay multiple egg masses, each containing from a few dozens to several thousands of eggs (García‐París, Montori, and Herrero 2004). The larval period generally spans for 2 or 3 months, occasionally more. Larvae feed on algae as well as plant and animal carrion. In turn, their most common predators are water snakes, turtles, fish, crayfish, diving beetles, and dragonfly naiads, among others (Egea‐Serrano 2014).

### 2.2 Study Area

Fieldwork took place in the southwest of Spain (37°20′ N, 7°09′ W) in two different habitats: Pinares de Cartaya and the agrosystems around it. Pinares de Cartaya is an extensive *Pinus pinea* grove (11,000 ha) whose underground is dominated by *Rosmarinus officinalis* , *Pistacia lentiscus,* and *Cistus ladanifer* . This formation might not be autochthonous, but it has been pervasive in the region for the last 4000 years at least, so it is interpreted as natural (Martínez and Montero 2004). On the other hand, the agrosystems around it encompass a traditionally extensive cropland, which has gradually shifted to intensive orange, strawberry, and raspberry plantations, among others. Agrochemicals are regularly added, especially via artificial irrigation systems, at owners' discretion. The areas sampled are about 6 km away from each other, which exceeds by several times the 49‐m average and the 250‐m maximum dispersal recorded for this species (Sánchez‐Montes and Martínez‐Solano 2011), while ensuring similar climatic conditions.

### 2.3 Experimental Design

In May 2023, we manually captured 10 individuals of each sex in each habitat. The individuals were sexed based on the presence of gray nuptial pads in the forelimbs of males, which also possess vocal sacs in their commissures that remain as dark skin creases when not in use; females lack these traits and tend to be larger (Egea‐Serrano 2014). Frogs were grouped according to the habitat of origin in two adjacent outdoor semi‐natural enclosures. These enclosures are 6 × 6 m long, with a 1‐m‐high brick wall from the ground on which a 1‐m‐high 5‐mm‐size rustproof steel mesh sits. The roof was covered with a mesh of the same type. Thus, frogs were unable to escape the enclosures, and predators were unable to enter them. Moreover, each enclosure had an 11‐m^2^, 50‐cm deep pond where frogs could mate (see the Supporting Information in Zambrano‐Fernández, Zamora‐Camacho, and Aragón 2020). The ponds were daily searched for egg masses. The egg masses detected were transferred to the laboratory within 12 h from oviposition. Immediately after reproduction, the parental frogs were released at their provenance habitats.

In total, we gathered 13 egg masses from agrosystem parents plus 19 egg masses from pine grove parents. Immediately upon collection, we randomly picked 15 eggs from each egg mass by spreading the egg mass in question on a tray and collecting eggs from different parts of it in no particular order. Each group of 15 eggs was placed in an experimental tank, a plastic aquarium (19 × 38 × 27 cm) with 6 L of untreated water, thus amounting to 32 experimental tanks and 480 eggs. These tanks were then allotted to one of two ammonium regimes. Half of these tanks, randomly selected, constituted the ammonium‐supplemented treatment. In these tanks, we added approximately 178.87 mg of 99.7% pure NH_4_Cl, leading to an ammonium (NH_4_^+^) concentration of 10 mg/L. According to previous research, a concentration of 13.5 mg NH_4_^+^/L provokes a mortality rate of 70% after 21 days of exposure in natural‐habitat *P. perezi* tadpoles (Egea‐Serrano, Tejedo, and Torralva 2009). The concentration we applied was slightly lower so as to avoid such mortality while still promoting sublethal effects (Zambrano‐Fernández, Zamora‐Camacho, and Aragón 2022). This concentration is environmentally relevant in Spain (Egea‐Serrano and Tejedo 2014; Garriga, Montori, and Llorente 2017). The remaining tanks constituted the non‐ammonium‐supplemented treatment, so NH_4_Cl was not added to them.

Besides, we assigned these tanks to one of two thermal regimes. To that end, in half of the aquaria from each ammonium regime, randomly selected, we included a 50‐W submergible heater (Marina Mini), which was connected continuously. These tanks constituted the heated treatment. The remnant tanks constituted the nonheated treatment, in which no artificial heating was implemented. Another complementary 32 tadpole‐free tanks were used, in which the thermal and ammonium regimes were applied as described above, but no tadpoles were added. These tanks served as a control of the physicochemical processes involved in the absence of tadpoles. The tadpole‐free tanks were subjected to the same treatments as the experimental tanks, but they were maintained only 4 days (the same time elapsed between water changes in the experimental tanks; see below), after which the temperature and ammonium concentration of all of them was measured with the same devices as the experimental tanks (see below).

The experimental tanks were kept on laboratory shelves. The room temperature in the laboratory was not controlled, but it fluctuated with the weather. The water was changed twice a week in the experimental tanks, but the treatments were maintained as described throughout the larval stage. In all cases, right before the water was changed, its temperature was measured in each tank to the nearest 0.1°C with a thermometer (OcioDual SKU: 80765), and the concentration of ammonium was also measured to the nearest 0.1 mg/L in a subsample of tanks of each ammonium regime with an Ammonia HR Checker (Hanna Instruments, HI733). Therefore, temperature and ammonium were measured two times a week. Following water changes, we randomly redistributed the tanks within the shelves to prevent potential slight differences in environmental conditions from affecting the tadpoles. Natural daylight entered the laboratory through a window, so that the animals could adjust their circadian rhythms. Tadpoles were fed boiled spinach *ad libitum*. Once a week, the number of survivors in each tank was registered.

Four weeks after hatching, before the earliest metamorphoses took place, we recorded swimming speed trials from above, using a camera Canon EOS 550D at 25 frames/s. To that end, the tadpoles were individually released in a white plastic tray (20 × 35 × 5 cm) filled with untreated natural water up to a depth of 4 cm. The water used was at about 18°C, which prevented differences in temperature from affecting locomotor performance (Perotti et al. 2018). After 2 min for habituation, the tadpoles were prompted to swim by gently prodding the base of their tails with a wooden stick. Each individual swam at least three times in a row before the recording was concluded. A ruler was included in each footage. Afterward, the resulting videos were analyzed with the software Tracker v.5.1.5. After calibrating distance against the ruler, the software identifies the position of the tadpole in consecutive frames, thus calculating the distance covered. As the time elapsed among frames is known, the speed of the tadpole between each pair of frames was calculated. For each individual, only the maximum speed reached was used in the analyses.

Immediately after the videos, having had excess water gently removed with a dry piece of absorbing paper, tadpoles were weighed to the nearest 0.01 g with a digital scale (CDS‐100). Then, they were laterally photographed with the same camera previously described, along with a piece of graph paper. The resulting photos were analyzed with the software ImageJ v.1.8.0. After calibrating distance against the graph paper, we obtained tadpole snout‐vent length (SVL). Also, we registered the Gosner stage of each individual as an indicator of the developmental phase (Gosner 1960).

The order in which the tadpoles were measured was random, and the process took 4 days. After these measurements, the tadpoles were returned to their tanks of origin, where they were kept in the aforesaid conditions in the tanks described until Gosner stage 42 when the tail begins to be resorbed. At that stage, tadpoles were transferred to tilted aquaria to facilitate transition to the terrestrial phase. Note that the tadpoles were not marked, as their small size could make the marking compromise their survival (Carlson and Langkilde 2013), so they could not be individually traced until metamorphosis. Metamorph locomotor performance was gauged on a polystyrene box (50 × 50 × 20 cm) in a room at around 18°C, which prevented differences in temperature from affecting locomotor performance (Navas, Gomes, and Carvalho 2008). Each froglet was individually released in one corner of the box, and a pin was stuck on the polystyrene right behind its hindquarters. Next, the frog was gently poked in the urostyle to stimulate jumping. Another pin was stuck behind its hindquarters on the spot of the box where it landed. The process was repeated five times. Then, the space between consecutive pins, tantamount to the jumping distance, was measured with a ruler. For each individual, we recorded the longest jump. The box was thoroughly rinsed with water among trials. Immediately after the trials, we measured the SVL of each individual to the nearest millimeter with a ruler and its body mass to the nearest 0.01 g with a digital scale (CDS‐100).

### 2.4 Statistical Analyses

Firstly, we tested that our experimental design, regarding the temperature and ammonium treatments, was successfully applied. Among the experimental tanks, water temperature was the response variable of a general linear mixed model (GLMM) where thermal regime (two levels: heated vs. nonheated), measurement sequence (the day of the experiment when the measurements in question were made), and their interaction were fixed factors and tank was a random factor. In the same way, ammonium concentration was the response variable of a mixed model where thermal regime, ammonium regime (two levels: with ammonium vs. without ammonium), measurement sequence, and their interaction were fixed factors and tank was a random factor. Among the tadpole‐free tanks, we conducted two separate GLMMs with tank as a random factor, one where water temperature was the response variable and thermal regime was a factor and another one where ammonium concentration was the response variable and thermal regime, ammonium regime, and their interaction were fixed factors.

Afterward, a GLMM was run where survivorship was the response variable, habitat (two levels: agrosystem vs. pine grove), ammonium regime, thermal regime, week, and their interactions were fixed factors and tank was a random factor. Then, we created a correlation matrix among the response variables measured for tadpoles (i.e., SVL, body mass, Gosner stage, and swimming speed; Table S1a) and another one among the response variables measured for metamorphs (i.e., SVL, body mass, days until metamorphosis, and jumping distance; Table S1b). In both cases, all variables were highly correlated (Table S1). Therefore, we performed two separate principal component analyses (PCAs), one for larvae and another one for metamorphs, including said variables, to condense them into fewer, uncorrelated principal components that avoid collinearity (Jongman, Braak, and Tongeren 1995). We applied the Guttmann–Kaiser criterion, according to which only principal components with an eigenvalue greater than 1 are considered (Yeomans and Golder 1982). Next, we conducted one GLMM per principal component considered, where the relevant principal component was the response variable, habitat, ammonium regime, thermal regime, and their interactions were fixed factors and tank was a random factor.

The assumptions of homoscedasticity and residual normality were checked (Quinn and Keough 2002), and, when not met even after data transformations, the function “*varIdent*” was applied (Zuur et al. 2009). The mixed models were conducted with the package “*nlme*” (Pinheiro et al. 2012) implemented in the software R (R Development Core Team 2012). In all cases, backward stepwise selection was applied. Significant interactions involving factors with two levels were further tested for pairwise comparisons with Tukey post hoc tests. Significant interactions involving factors with more than two levels were analyzed visually for the sake of simplicity. The full models prior to backward stepwise selection, along with the results of the Tukey post hoc tests, are presented in the Appendix S1 and S2.

## 3 Results

### 3.1 Water Temperature and Ammonium Concentration

**FIGURE 1:**
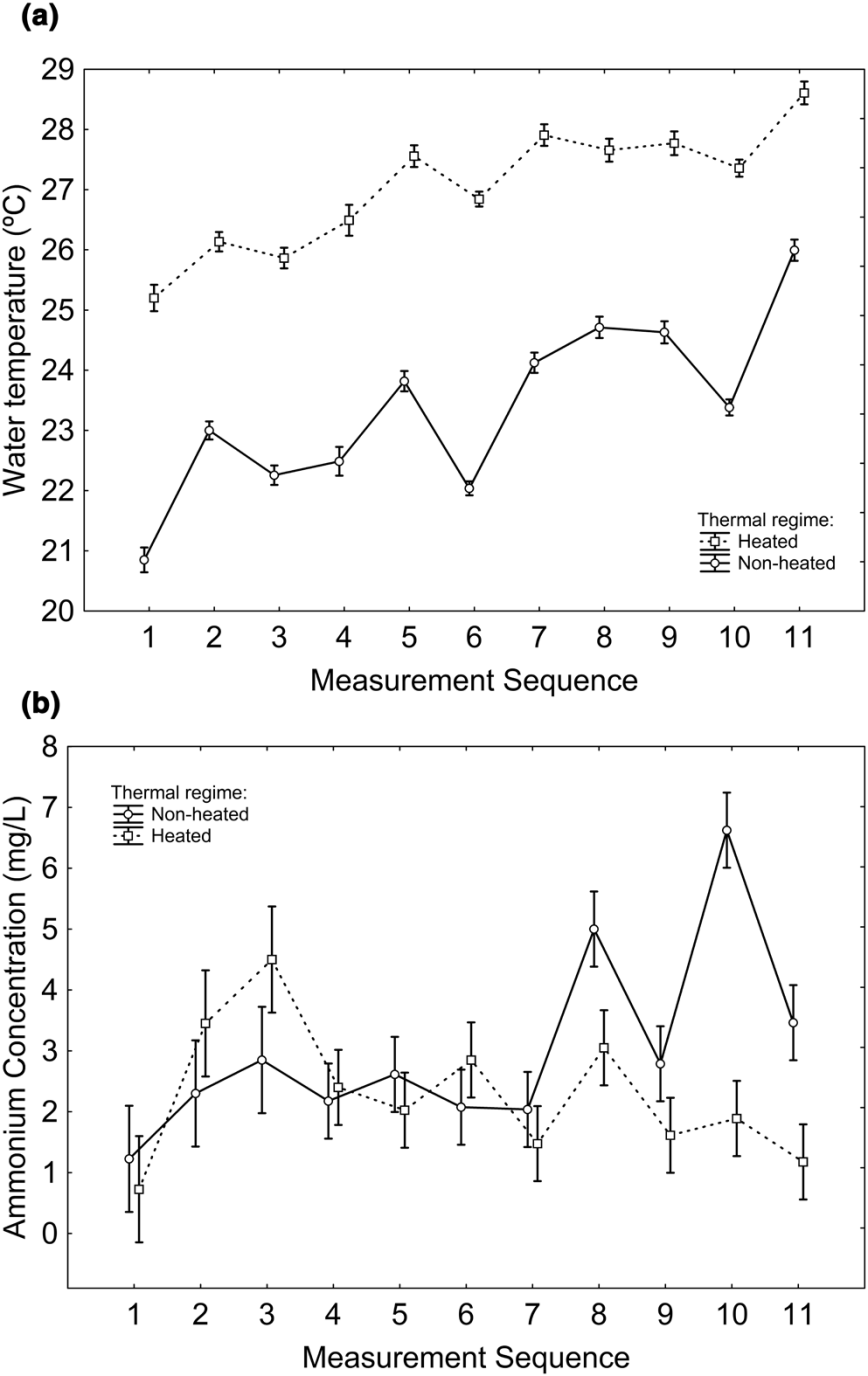
Water temperature (a) and ammonium concentration (b) in heated and nonheated tanks along subsequent biweekly measurements starting in late May and finishing in early July 2023. Vertical whiskers indicate standard errors. The number of tanks measured was 16 per thermal regime.

Among the experimental tanks (where the tadpoles were raised), water temperature was greater in heated tanks (*Χ* ^2^_1,30_ = 315.488; *p* < 0.001; Figure 1a), tended to increase in subsequent measurements (*Χ* ^2^_1,315_ = 264.903; *p* < 0.001; Figure 1a), and the interaction thermal regime × measurement sequence was significant (*Χ* ^2^_1,315_ = 5.431; *p* = 0.020; Figure 1a), where the pattern reflects fluctuations that did not overlap between the two thermal regimes (Figure 1a). Ammonium concentration was greater in ammonium‐supplemented tanks (*Χ* ^2^_1,29_ = 57.663; *p* < 0.001; Figure 1b), in nonheated tanks (*Χ* ^2^_1,29_ = 6.136; *p* = 0.014; Figure 1b), and varied according to the day of measurement (*Χ* ^2^_1,118_ = 19.577; *p* < 0.001; Figure 1b) and to the interaction thermal regime × day of measurement (*Χ* ^2^_1,118_ = 18.851; *p* < 0.001; Figure 1b).

Among the tadpole‐free tanks (where no tadpoles were housed), water temperature was greater in heated tanks [mean ± SE (°C); nonheated: 21.225 ± 0.239; heated: 26.831 ± 0.239; *Χ* ^2^_1,30_ = 274.650; *p* < 0.001], and ammonium concentration was greater in ammonium‐supplemented tanks [mean ± SE (mg/L); nonsupplemented: 0.344 ± 0.221; supplemented: 12.238 ± 0.221; *Χ* ^2^_1,30_ = 1453.564; *p* < 0.001], whereas the effect of thermal regime on ammonium was nonsignificant.

### 3.2 Survivorship

**FIGURE 2:**
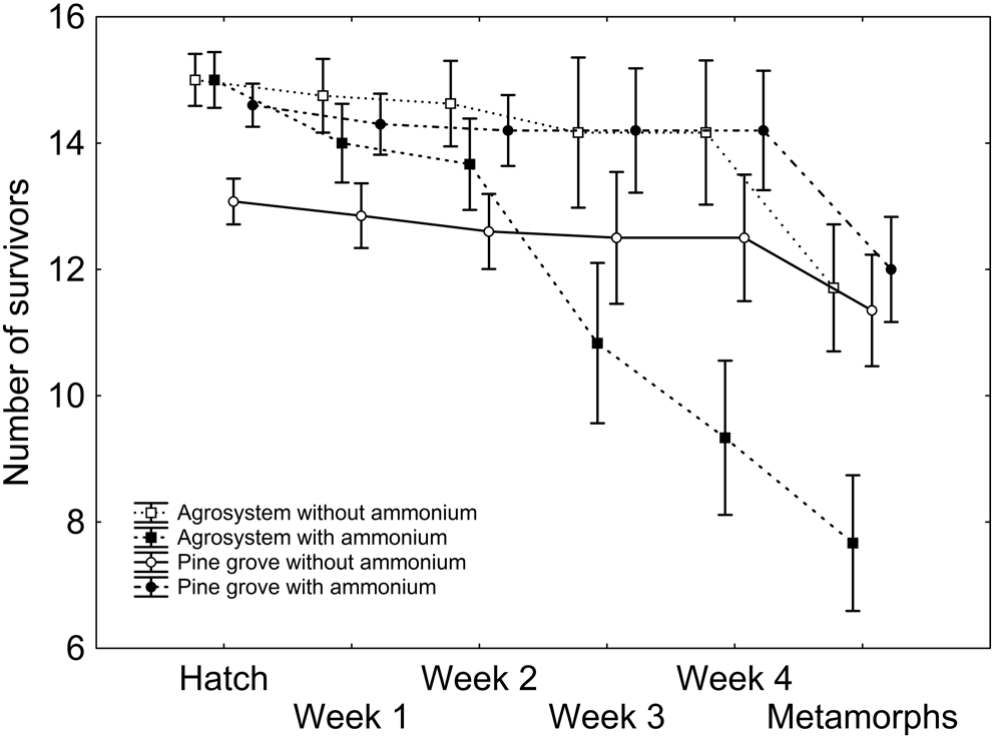
Number of survivors per tank according to counting week, habitat, and ammonium regime. Squares represent agrosystem individuals, whereas circles represent pine grove individuals. Empty symbols represent non‐ammonium‐supplemented tanks, whereas solid symbols represent ammonium‐supplemented tanks. Vertical whiskers represent standard errors.

After backward stepwise selection was applied to the full model where survivorship was the response variable (Table S2), significant effects of week (*Χ* ^2^_5,120_ = 26.211; *p* < 0.001; Figure 2), the two‐way interaction ammonium regime × week (*Χ* ^2^_5,120_ = 34.341; *p* < 0.001; Figure 2), and the three‐way interaction ammonium regime × week × habitat (*Χ* ^2^_5,120_ = 19.207; *p* = 0.002; Figure 2) remained, according to which survivorship tended to diminish in time, significantly more in agrosystem tadpoles subjected to ammonium contamination (Figure 2).

### 3.3 Larvae

#### 3.3.1 Principal Component Analysis

Only the first principal component (PC1‐L), which explained 67.766% of the total variance, had an eigenvalue greater than 1 (2.711). PC1‐L was negatively correlated with SVL, body mass, Gosner stage, and swimming speed (Table S3a).

#### 3.3.2 Mixed Model

**FIGURE 3:**
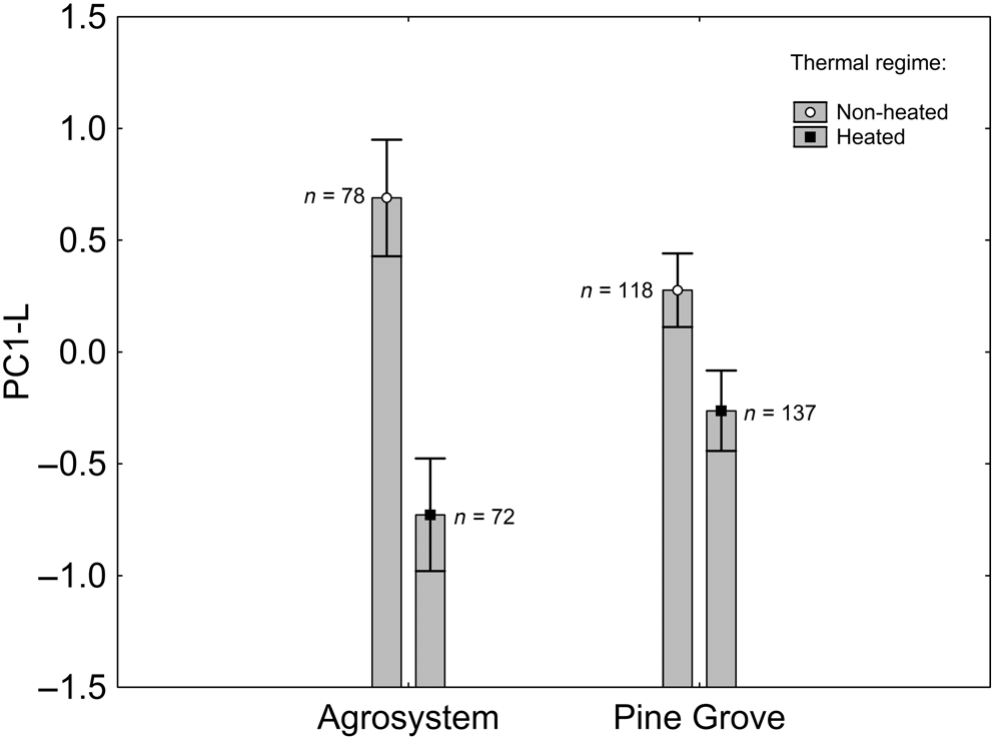
PC‐L (principal component negatively correlated with larva SVL, body mass, Gosner stage, and swimming speed) according to the habitat and thermal regime of tadpoles. Sample sizes are indicated. Vertical whiskers represent standard errors.

The full model where larva PC1‐L was the response variable is available in the Table S4a. The final model, after backward stepwise selection, included a nonsignificant effect of habitat (*Χ* ^2^_1,401_ = 1.796; *p* = 0.180; Figure 3), significant effects of thermal regime (*Χ* ^2^_1,401_ = 15.362; *p* < 0.001; Figure 3), and the thermal regime × habitat interaction (*Χ* ^2^_1,401_ = 4.047; *p* = 0.044; Figure 3). According to the Tukey post hoc test applied on the interaction (Table S5), PC‐L was greater in nonheated than in heated individuals from the agrosystem, which was not true for pine grove individuals. In other words, SVL, body mass, Gosner stage, and swimming speed were greater in heated than in nonheated agrosystem individuals, whereas thermal regime did not affect pine grove individuals.

### 3.4 Metamorphs

#### 3.4.1 Principal Component Analysis

Only the first principal component (PC1‐M), which explained 77.133% of the total variance, had an eigenvalue greater than 1 (3.085). PC1‐M was negatively correlated with SVL, body mass, days until metamorphosis, and jumping distance (Table S3b).

#### 3.4.2 Mixed Model

The full model where larva PC1‐M was the response variable is available in the Table S4b. The final model, after backward stepwise selection, included only the significant effects of temperature, according to which PC‐M was greater in metamorphs reared in heated tanks than in those reared in nonheated tanks (mean ± standard error; nonheated: −0.597 ± 0.148; heated: 0.562 ± 0.146; *Χ* ^2^_1,341_ = 31.868; *p* < 0.001) and of habitat, according to which PC‐M was greater in metamorphs from pine grove than in those from agrosystem (mean ± standard error; agrosystem: −0.810 ± 0.200; pine grove: 0.478 ± 0.119; *Χ* ^2^_1,341_ = 30.478; *p* < 0.001). In other words, SVL, body mass, days until metamorphosis, and jumping distance were greater in metamorphs reared in nonheated tanks than in those reared in heated tanks. These variables were also greater in agrosystem than in pine grove metamorphs.

## 4 Discussion

The analyses conducted on the tadpole‐free tanks demonstrate that the experimental design was efficient in creating two different thermal and ammonium regimes in a noninteractive way, so that temperature did not affect the concentration of ammonium. The greater concentration of ammonium in nonheated experimental tanks at the latest stages of the experiment might be a consequence of tadpole metabolism. In fact, the amount and proportion of excreted ammonia and urea in tadpoles are dependent on temperature and the developmental stage (Ashley, Katti, and Frieden 1968).

Temperature had a major effect on larval development. As predicted, and as found in other anurans (Álvarez and Nicieza 2002a; Yu and Han 2020; Park, Park, and Do 2023), larvae in heated tanks developed faster and metamorphosed earlier, which could be a consequence of the temperature increasing the standard metabolic rates (Ruthsatz et al. 2018), triggering the expression of genes (Chen et al. 2021; Fan et al. 2021), and the cascade of thyroid hormones (Hammond, Veldhoen, and Helbing 2015; Suzuki et al. 2016) that govern the process of metamorphosis. If high temperatures function as a stressor, exposure to it might be minimized by a hormone‐mediated acceleration of development (Denver 2009). However, it should be noted that larvae reared in heated water also grew faster, although only in the case of agrosystem individuals. The effects of temperature on the larval body size can be species‐specific (Maciel and Juncá 2009) and vary geographically even within the same species, with tadpoles from warmer habitats growing more when reared in warmer water (Olsson and Uller 2002). On the whole, there seems to be an optimal temperature that maximizes growth, which varies from species to species, above and below which growth tends to diminish (Harkey and Semlitsch 1988; Maciel and Juncá 2009; Fan et al. 2021; Ren et al. 2021). In this case, *P. perezi* is a thermophile species (Egea‐Serrano 2014). As such, the temperature in heated tanks might not exceed its thermal optimum, while that in nonheated tanks appears to be below it. Aligned with this reasoning, the swimming speed of tadpoles in the heated treatment was also higher, which could be a consequence of temperature favoring ossification (Ren et al. 2021) or muscle fiber recruitment in the tail (Arendt and Hoang 2005). It should also be noted that the sex of the tadpoles, which can determine the physiological consequences of temperature (Phuge 2017), was unknown and thus not controlled for.

In the case of metamorphs, those resulting from nonheated tanks were larger, which could be a byproduct of a more prolonged larval—and therefore growth—period, and is along the lines of previous research where tadpoles at lower temperatures metamorphosed later but at greater body sizes (Harkey and Semlitsch 1988; Álvarez and Nicieza 2002a; Phuge 2017; Yu and Han 2020). This is particularly relevant considering the fact that size at metamorphosis may predict the subsequent survival and growth in some anurans (Cabrera‐Guzmán et al. 2013; Székely et al. 2020). Also, the jumping distance tended to be greater in metamorphs reared in nonheated tanks. This is aligned with some previous research (Álvarez and Nicieza 2002b) but contrasts with other works where temperature during the larval period did not affect locomotor performance of metamorphs (Beck and Congdon 2000).

The overriding consequences of temperature on both larvae and metamorphs could be masking any potential effects of ammonium. In fact, the effects of ammonium were negligible in the traits measured in these animals. This is in stark contrast with former findings in this study system, where exposure to the same concentration of ammonium used here (but where temperature was not altered) gave way to larvae with a faster development, an increased body size and mass, and a hindered swimming performance in pine grove individuals (Zambrano‐Fernández, Zamora‐Camacho, and Aragón 2022). Nonetheless, most of those effects disappeared in the metamorphs that those tadpoles produced, although the jumping performance was likewise negatively affected by ammonium in pine grove individuals (Zamora‐Camacho, Zambrano‐Fernández, and Aragón 2022), which was aligned with the result found in larvae (Zambrano‐Fernández, Zamora‐Camacho, and Aragón 2022) but not with those found in this piece of research involving a manipulation of temperature as well as ammonium concentration.

Interestingly, habitat affected the response of these frogs to the thermal and ammonium regimes undergone. In the case of larvae, while neither pine grove nor agrosystem individuals were affected by ammonium, pine grove individuals proved less sensitive to the thermal regime. In past investigations conducted on this system, the hypothesis that agrosystem individuals would be less sensitive to ammonium has also received little support (Zambrano‐Fernández, Zamora‐Camacho, and Aragón 2022; Zamora‐Camacho, Zambrano‐Fernández, and Aragón 2022). In turn, agrosystem metamorphs in this work were heavier, took longer to complete their metamorphoses, and exhibited greater jumping distance, which is aligned with previous findings in this system (Zamora‐Camacho, Zambrano‐Fernández, and Aragón 2022). According to other studies, the sensitivity of the locomotor performance of anuran metamorphs to the thermal conditions during the larval stage may vary at a geographical (Drakulić et al. 2016), even microgeographical (Orizaola and Laurila 2009), level.

## 5 Conclusion

To conclude, this research is novel to highlight how the effects of temperature can override any potential effects of ammonium in anuran tadpoles and the metamorphs they give rise to. Increased temperature had major effects on these animals, leading to an acceleration in development and metamorphosis. Increased water temperature potentiated tadpole growth while producing smaller metamorphs. The former could be a consequence of increased metabolism, whereas the latter could result from a reduced larval, and therefore growth, period. At the same time, swimming speed was greater in tadpoles reared in heated tanks, while jumping distance was greater in metamorphs reared in nonheated tanks. However, the effect of temperature was more marked in agrosystem than in pine grove individuals, which could mirror a reduced adaptability due to hampered connectivity. Moreover, the ability to buffer the effects of temperature diverges locally, being lower in the agrosystem. Future research avenues for a better understanding of the global change impacts on biodiversity may include further experimental designs involving other global change factors in the short, mid‐, and long terms.

## Author Contributions

**Francisco Javier Zamora‐Camacho:** conceptualization (equal), formal analysis (equal), investigation (equal), methodology (equal), writing – original draft (equal). **Pedro Aragón:** conceptualization (equal), investigation (equal), project administration (equal), supervision (equal), validation (equal), writing – review and editing (equal).

## Conflicts of Interest

The authors declare no conflicts of interest.

## Supporting information


Appendix S1.

Appendix S2.

## Data Availability

The dataset is provided in the Appendices S1 and S2.
