# Supplementary material for: Increased Temperature and Exposure to Ammonium Alter the Life Cycle of an Anuran Species
Source: Ecol Evol. 2024 Dec 2;14(12):e70685. doi: 10.1002/ece3.70685 (PMC11612019; doi:10.1002/ece3.70685)
Supplement: Supplementary file 2 — Appendix S2. [file ECE3-14-e70685-s001.docx]

**Increased temperature and exposure to ammonium alter the life cycle of an anuran species**

**Supplementary Material**

Table S1 – Correlation matrices among the response variables measured for tadpoles (i.e., SVL, body mass, Gosner stage and swimming speed; Table S1a) and metamorphs (i.e., SVL, body mass, days until metamorphosis and jumping distance; Table S1b). Symbols indicate: *** = *P* < 0.001. Significant results are in bold.

|  | **SVL** | **Body Mass** | **Gosner Stage** | **Swimming Speed** |
| --- | --- | --- | --- | --- |
| **SVL** |  | **0.898***** | **0.733***** | **0.408***** |
| **Body Mass** | **0.898***** |  | **0.602***** | **0.370***** |
| **Gosner Stage** | **0.733***** | **0.602***** |  | **0.264***** |
| **Swimming Speed** | **0.408***** | **0.370***** | **0.264***** |  |

Table S1a

|  | **SVL** | **Body Mass** | **Days until Metamorphosis** | **Jumping Distance** |
| --- | --- | --- | --- | --- |
| **SVL** |  | **0.928***** | **0.677***** | **0.631***** |
| **Body Mass** | **0.928***** |  | **0.696***** | **0.677***** |
| **Days until Metamorphosis** | **0.677***** | **0.696***** |  | **0.531***** |
| **Jumping Distance** | **0.631***** | **0.677***** | **0.531***** |  |

Table S1b

Table S2 – Full model with survivorship as the response variable and habitat, ammonium regime, thermal regime, week, and their interactions were included as factors. Degrees of freedom (DF), *Χ*^2^-values and *P*-values are shown. Significant results are in bold.

| **Variable** | **DF** | ***Χ*^2^** | ***P*-value** |
| --- | --- | --- | --- |
| **Habitat** | 1, 24 | 1.887 | 0.170 |
| **Ammonium Regime** | 1, 24 | <0.001 | 1.000 |
| **Thermal Regime** | 1, 24 | <0.001 | 1.000 |
| **Week** | **5, 120** | **12.243** | **0.032** |
| **Habitat*Ammonium Regime** | 1, 24 | 0.491 | 0.484 |
| **Habitat*Thermal Regime** | 1, 24 | 0.076 | 0.783 |
| **Ammonium Regime*Thermal Regime** | 1, 24 | <0.001 | 1.000 |
| **Habitat*Week** | 5, 120 | 0.654 | 0.985 |
| **Ammonium Regime*Week** | **5, 120** | **24.071** | **<0.001** |
| **Temperature*Week** | 5, 120 | 0.399 | 0.995 |
| **Habitat*Ammonium Regime*Thermal Regime** | 1, 24 | 0.006 | 0.941 |
| **Habitat*Ammonium Regime*Week** | **5, 120** | **13.408** | **0.020** |
| **Habitat*Thermal Regime*Week** | 5, 120 | 0.694 | 0.983 |
| **Ammonium Regime*Thermal Regime*Week** | 5, 120 | 3.494 | 0.624 |
| **Habitat*Ammonium Regime*Thermal Regime*Week** | 5, 120 | 2.740 | 0.740 |

Table S3 – Correlations between the first principal components either for larvae (PC1-L, Table S3a) or metamorphs (PC1-M, Table S3b) and the variables included in the relevant Principal Component Analyses.

| **Variable** | ***r*-value** |
| --- | --- |
| **SVL** | -0.958 |
| **Body Mass** | -0.909 |
| **Gosner Stage** | -0.815 |
| **Swimming Speed** | -0.551 |

Table S3a

| **Variable** | ***r*-value** |
| --- | --- |
| **SVL** | -0.932 |
| **Body Mass** | -0.949 |
| **Days until Metamorphosis** | -0.823 |
| **Jumping Distance** | -0.800 |

Table S3b

Table S4 – Full model where either PC1-L (Table S4a) or PC1-M (Table S4b) were the response variables. *Χ*^2^ and *P*-values are indicated. Significant results are in bold.

| **Variable** | ***Χ*^2^_1, 397_** | ***P*-value** |
| --- | --- | --- |
| **Ammonium regime** | 1.410 | 0.235 |
| **Thermal regime** | 1.544 | 0.214 |
| **Habitat** | 0.071 | 0.790 |
| **Ammonium regime*Thermal regime** | 2.853 | 0.091 |
| **Ammonium regime*Habitat** | 1.233 | 0.267 |
| **Thermal regime*Habitat** | 0.283 | 0.594 |
| **Ammonium regime*Thermal regime*Habitat** | 2.281 | 0.131 |

Table S4a

| **Variable** | ***Χ*^2^_1, 336_** | ***P*-value** |
| --- | --- | --- |
| **Ammonium regime** | 1.067 | 0.302 |
| **Thermal regime** | **9.374** | **0.002** |
| **Habitat** | **10.011** | **0.002** |
| **Ammonium regime*Thermal regime** | 0.435 | 0.510 |
| **Ammonium regime*Habitat** | 1.320 | 0.251 |
| **Thermal regime*Habitat** | 0.346 | 0.556 |
| **Ammonium regime*Thermal regime*Habitat** | 0.953 | 0.329 |

Table S4b

Table S5 – Tukey post-hoc test applied on the significant temperature*habitat interaction for the model were PC1-L was the response variable. *t*- and *P*-values are indicated. Significant results are in bold.

| **Pairwise comparison** | ***t*_1,401_** | ***P*-value** |
| --- | --- | --- |
| **Non-heated Agrosystem-Heated Agrosystem** | **3.919** | **0.003** |
| **Non-heated Agrosystem-Non-heated Pine Grove** | 1.340 | 0.546 |
| **Non-heated Agrosystem-Heated Pine Grove** | **3.011** | **0.027** |
| **Heated Agrosystem-Non-heated Pine Grove** | **-3.342** | **0.012** |
| **Heated Agrosystem-Heated Pine Grove** | -1.504 | 0.448 |
| **Non-heated Pine Grove-Heated Pine Grove** | 2.210 | 0.145 |
